# Supplementary material for: Content-rich biological network constructed by mining PubMed abstracts
Source: BMC Bioinformatics. 2004 Oct 8;5:147. doi: 10.1186/1471-2105-5-147 (PMC528731; doi:10.1186/1471-2105-5-147)
Supplement: Additional File 5 — The original Chilibot query results of the term "long-term potentiation (LTP)" and 22 other terms, limiting the latest references analyzed to the years 1990, 1995, 2000, and 2004. [file 1471-2105-5-147-S5.bz2 › chilibotAdditionalFile5/ltp1995/html/CREB_PI-3K.html]

 


 **CREB** and **PI-3K** 
  
Found 1 abstracts in PubMed,  **1 abstracts were retrieved and analyzed**.  


---

 Search Google  |
 PDF files only 
|  EDU domain only 

---

- NatureNature,   **Inhibition of glycogen synthase kinase 3 by insulin mediated by protein kinase B.** .
  Glycogen synthase kinase 3 GSK3 is implicated in the regulation of several physiological processes, including the control of glycogen and protein synthesis by insulin, modulation of the transcription factors AP 1 and **CREB**, the specification of cell fate in Drosophila and dorsoventral patterning in Xenopus embryos.
  GSK3 is inhibited by serine phosphorylation in response to insulin or growth factors and in vitro by either MAP kinase activated protein MAPKAP kinase 1 also known as p90rsk or p70 ribosomal S6 kinase p70S6k .
  Here we show that agents which prevent the activation of both MAPKAP kinase 1 and p70S6k by insulin in vivo do not block the phosphorylation and inhibition of GSK3.
  Another insulin stimulated protein kinase inactivates GSK3 under these conditions, and we demonstrate that it is the product of the proto oncogene protein kinase B PKB, also known as Akt RAC .
  Like the inhibition of GSK3 refs 1 4 , the activation of PKB is prevented by inhibitors of phosphatidylinositol PI 3 kinase **[PI-3K]**.
